# Substantial Genetic Progress in the International *Apis mellifera carnica* Population Since the Implementation of Genetic Evaluation

**DOI:** 10.3390/insects11110768

**Published:** 2020-11-07

**Authors:** Andreas Hoppe, Manuel Du, Richard Bernstein, Friedrich-Karl Tiesler, Martin Kärcher, Kaspar Bienefeld

**Affiliations:** 1 Institute for Bee Research Hohen Neuendorf, Friedrich-Engels Str. 32, 16540 Hohen Neuendorf, Germany; manuel.du@hu-berlin.de (M.D.); richard.bernstein@hu-berlin.de (R.B.); kaspar.bienefeld@hu-berlin.de (K.B.); 2 Albrecht Daniel Thaer-Institute for Agricultural and Horticultural Sciences, Humboldt University of Berlin, 10099 Berlin, Germany; 3 Deutscher Imkerbund e.V., Villiper Hauptstraße 3, 53343 Wachtberg, Germany; fk.tiesler@tiesler-bau.de; 4 Austrian Carnica Association, Leonhardstraße 114/35, 8010 Graz, Austria; martin_kaercher@yahoo.de

**Keywords:** honeybee, *A. m. carnica*, breeding, BLUP, breeding value, BeeBreed.eu

## Abstract

**Simple Summary:**

The *Apis mellifera carnica* subspecies of the honeybee is known for its gentleness and good honey yield. In the early 20th century, systematic breeding efforts began. Breeding progress was slow before the introduction of modern techniques of genetic evaluation in the mid 1990s. Here, the results of the official breeding value estimation in BeeBreed.eu are analyzed. From about 2000 onward, breeding progress accelerated. The result is a considerable gain in honey yield and desirable properties without increased inbreeding coefficients. The success of *A. m. carnica* breeding shows the potential of genetic evaluation.

**Abstract:**

The *Apis mellifera carnica* subspecies of the honeybee has long been praised for its gentleness and good honey yield before systematic breeding efforts began in the early 20th century. However, before the introduction of modern techniques of genetic evaluation (best linear unbiased prediction, BLUP) and a computerized data management in the mid 1990s, genetic progress was slow. Here, the results of the official breeding value estimation in BeeBreed.eu are analyzed to characterize breeding progress and inbreeding. From about the year 2000 onward, the genetic progression accelerated and resulted in a considerable gain in honey yield and desirable properties without increased inbreeding coefficients. The prognostic quality of breeding values is demonstrated by a retrospective analysis. The success of *A. m. carnica* breeding shows the potential of BLUP-based breeding values and serves as an example for a large-scale breeding program.

## 1. Introduction

*Apis mellifera carnica*, the Carniolan bee, originating from an area roughly bordered by the Alps in the northwest, the Carpathian Mountains in the northeast, the Mediterranean Sea in the southwest and the Albanian Alps in the south [1], has attracted the attention of many beekeepers worldwide [2]. In addition to their original distribution area, the Carnica is probably the most commonly kept subspecies in most Central and Northern European countries [3,4,5]. The distribution of *A. m. carnica* bees began in the 18th century from the Carniolan region, now Slovenia [1], and the rapid multiplication of colonies in the 19th century with swarms [6] had the side effect that colonies were selected for stronger swarming drive. Systematic breeding efforts [7] by Sklenar, Wrisnig and Peschetz in Austria in the 1930s led to honeybees less prone to swarming and adapted to the local environments [1] which formed the basis for *A. m. carnica* breeding in Austria, Germany and Switzerland after 1945, evolving into the international breeding program which is the topic of this paper.

As mating control is essential for sustained breeding progress [8], mating stations have been established as early as in 1870 in Hroby near Tábor (Austria–Hungary, now Czech Republic) by Rudolph Kolowrat [9]. The first mating station in Germany was Ohrwaschl in 1908 [10] while the oldest still active mating station is Gehlberg, established in 1911 [11]. The discovery of multiple mating in 1954 [12,13] led to the design of mating stations where multiple drone-producing colonies are headed by daughters of the same colony [14], a setup still used today.

The traditional breeding aims in the *A. m. carnica* breeding program are (i) honey yield (as an economic factor but also an indication of a strong and healthy colony [15]) and manageability which includes (ii) gentleness, reduced defensive behavior against humans, (iii) calmness on the comb which simplifies the inspection process, and (iv) swarming, i.e., reduced swarming drive, which facilitates the beekeeper’s interactions to avoid swarming. These four traits form the classical set of breeding objectives considered since the early 20th century, first in Austria and Germany, and subsequently in countries where these breeds continued [16].

To reduce disease susceptibility is an important breeding goal, in particular for the resistance against *Varroa destructor* [17] and their related diseases [18,19,20]. Therefore, *Varroa* resistance is recognized as a critical breeding goal [21,22], and brood hygiene, measured by the pintest (counting how many cells have been cleared after larvae have manually been killed with a pin), mite fall in spring, and mite measurements in summer form the *Varroa*-related breeding traits [23]. Hygienic behavior is generally found to be beneficial for *Varroa* resistance [24] although this is controversially discussed [25].

Starting from a recollection of data from the archives of several regions in West Germany (1988) and the GDR (1990), the database BeeBreed [26] has developed into a registry of most queens involved in large-scale breeding programs in Europe.

Henderson’s best linear unbiased prediction (BLUP) [27] provides a method of genetic evaluation based on the relationships between animals in the population. It was soon widely applied in livestock breeding, leading to significant genetic gain (e.g., [28,29]).

The general breeding value model applied for the BeeBreed-hosted breeding value estimation has been reported before [30,31] and will not be detailed here as opposed to [32,33,34]. Data acquisition is based on the Apimondia guidelines for the performance testing of honeybees [16]. It includes the complex paternal descent [35], considers direct and maternal effects [36], and uses multi-trait models for appropriate traits [37]. The relationships used for BLUP are based on a probabilistic relationship calculation [38,39]. Model design and parameters are refined frequently after careful validation [40].

Here, we report the genetic progress of the current *A. m. carnica* breeding population and other properties of the breeding values.

## 2. Materials and Methods

### 2.1. Data Resource

Breeding value estimation is based on the performance tests of 195,458 queens of the *A. m. carnica* population. The (mated) queen is tested by the properties of its colony—this wording is traditional to the breeding community, and followed throughout the manuscript. To complete the pedigree, ancestry information of further queens are added as additional datasets (line 2 in Table 1). Some queens are only referenced as a mother or mating ancestor of another queen (line 1 in Table 1) giving a total of 224,081 queens for which breeding values have been estimated on 14 February 2020.

The data were deposited in the online service BeeBreed [26] and span over 12 countries (Table 2). Breeding material is frequently exchanged between countries to a varying degree. It is a connected population, although parts of the population are relatively separate, such as the Swedish or Swiss population. Breeding for *Varroa* resistance as an international effort often crosses nation borders while some breeder groups aim to preserve breeding lines for environmental adaptation or particular traits.

The trait honey refers to the total honey yield in kilograms including reserve left in the hive. The behavioral traits of gentleness, calmness, and swarming tendency were evaluated by marks on a scale from 1 to 4, where 1 is worst and 4 is best [41,42]. While “gentleness” refers to the aggressivity toward the beekeeper and comprises stinging and menacing flight, “calmness” describes the steadiness of workerbees on the comb. Although similar and often coincident, both properties are clearly distinguishable. The evaluation of swarming tendency not only includes the swarming itself but also the signs of preparation, e.g., the construction of swarming cells and unrest of house bees.

Hygienic behavior is assessed by a specific test in which the brood is killed either with a pin or by freezing as the percentage of cells with removed brood after a waiting period. Recently, the protocol has been modified to count opened cells [43] but the breeding data used here is based on the previous protocol.

The *Varroa* infestation development (VID) is assessed by mite infestation measurements in summer and the natural mite fall measured in spring. For a full account of the performance test, see [41,42,44]. In the majority of the colonies where *Varroa* infestation is reported, it is only a single measurement without a given date. Typically, it is taken at the 27th calender week. For colonies with more than one measurement, each measurement is adjusted to the 27th calendar week and then averaged. The adjustment uses a function obtained from an exponential regression of the multiple infestation data within the breeding data.

The natural mite fall measurement in spring represents the starting condition of the colony. Thus, an adjustment term resulting from a regression is subtracted. The average of the adjusted mite infestation measurements minus the starting condition term is the final trait value for VID,

v=1n∑i=1nbie(186.5−di)/56−2.0ln(1+M)−0.600if M measuredelsemitesper10gbeesample,

where *M* is natural mite fall in spring given in mites per day (unit removed), *n* is the number of infestation measurements in summer, $b_{i}$ is the *i*-th infestation measurements in mites per 10 g bee sample, and $d_{i}$ is the calendar day of the year of the *i*-th infestation measurement. The unit of the trait is “mites per 10 g bee sample”, and in the case of a single infestation measurement in the middle of 27th week with no measured mite fall the trait value equals the measurement. The adjustment term for mite fall is calibrated (by the −0.60 term) so that in case of a missing measurement the average mite fall is assumed.

The data are refined with the following procedures:

- trait data are removed when in a specific apiary/season all colonies have the same values (because equality tends to produce artifacts in multi-trait models),
- for honey yield or VID, a value outside 4-times standard deviation of an apiary/season’s values is considered as an outlier and compressed towards the apiary’s average [45] because outliers “are likely to fail to fit the model” [46] and negatively impact breeding value estimation, and
- the values on an apiary/season are condensed or expanded to align the phenotypical variances on the respective apiaries, similarly to breeding programs of other farm animals [47].

### 2.2. Relationships and Models

Using the queen’s studbook information [44], a pedigree is set up with the following items:

- queen,
- worker community,
- pseudo sire (“Pseudo” refers to the fact that it is not a single animal but a group of animals, more details can be found in [39,48]) which is either (i) a run of a mating station, (ii) an insemination run with a common set of drone producing colonies, or (iii) an apiary environment in a specific season,
- potential offspring queen.

PInCo [39] is used to calculate the inverse relationship matrix of all pedigree items for direct input in implementation BLUPF90 [49] of the breeding value estimation.

A colony’s trait value y is represented as the sum of one random effect of the queen $Z_{Q}a_{Q}$, a second random effect of the worker community $Z_{W}a_{W}$, one fixed effect Xb (specific for the test location and the test year), and a residual effect e, in a mixed-linear additive genetic model [50] as:

$$y=Xb+Z_{W}a_{W}+Z_{Q}a_{Q}+e,$$

where $Z_{Q}$ and $Z_{W}$ are the relationship matrices and $a_{Q}$ and $a_{W}$ are the breeding values of queen and workers, respectively.

For the calculation of breeding values, two separate models were used: a 4-trait-model for the classical traits honey yield, gentleness, calmness and swarming, and a 2-trait model for the *Varroa*-related traits hygienic behavior and VID. The split of traits was dictated by the results of model validation [40] with the likely explanation that both *Varroa*-related traits were only recorded in about 15% of all performance tests and 55% of the current population. The heritabilities and genetic correlations of the models are given in Table 3.

The definition of heritabilities in honeybee breeding requires nuanced consideration [51], as the workers are a community of genetically heterogeneous diploid animals. The phenotypical variance is calculated as $\sigma_{P}^{2}=a_{SS}\sigma_{A^{W}}^{2}+\sigma_{A^{Q}}^{2}+\sigma_{A^{Q}A^{W}}+\sigma_{E}^{2}$ [51], where the variances $\sigma_{A^{W}}^{2}$, $\sigma_{A^{Q}}^{2}$, $\sigma_{A^{Q}A^{W}}$, and $\sigma_{E}^{2}$ are the variance components of the model fitted with AIREMLF90 [49]. The additive genetic relationship $a_{SS}$ between two drone producing queens (DPQ) reared from the same colony [51] was calculated as $a_{SS}=0.41136$ using PInCo [39]. The heritability of the queen effect is $h_{Q}^{2}=\frac{\sigma_{A^{Q}}^{2}}{\sigma_{P}^{2}}$, the heritability of the worker effect is $h_{W}^{2}=\frac{a_{SS}\sigma_{A^{W}}^{2}}{\sigma_{P}^{2}}$, and the heritability of the selection criterion is $h_{SC}^{2}=\frac{\sigma_{A^{W}}^{2}+\sigma_{A^{Q}}^{2}+2\sigma_{A^{Q}A^{W}}}{\sigma_{P}^{2}}$. The accessible heritability is defined as $h_{A}^{2}=a_{SS}h_{SC}^{2}$. It refers to offspring queens which are randomly chosen from the colony. In comparison to livestock, like cattle, the response to selection is lowered by the specifics in bee parentage because the offspring queen itself is not subjected to the performance test but the workers (half-sisters of the offspring queen and half-cousins at the same time). The genetic variability is accounted for in the approximation of accessible heritability $h_{A}^{2}$.

See Table 4 for genetic correlations between the traits as relevant for the model. As there is a separate model for the four classical traits and the two *Varroa* traits, only the correlations within the four classical traits and between the two *Varroa* traits are given.

The genetic correlation between two traits is calculated as

$$\frac{\sigma_{A_{1}^{W}A_{2}^{W}}+\sigma_{A_{1}^{W}A_{2}^{Q}}+\sigma_{A_{2}^{W}A_{1}^{Q}}+\sigma_{A_{1}^{Q}A_{2}^{Q}}}{\sqrt{\left(\sigma_{A_{1}^{W}}^{2}+\sigma_{A_{1}^{Q}}^{2}+2\cdot \sigma_{A_{1}^{W}A_{1}^{Q}}\right)\cdot \left(\sigma_{A_{2}^{W}}^{2}+\sigma_{A_{2}^{Q}}^{2}+2\cdot \sigma_{A_{2}^{W}A_{2}^{Q}}\right)}},$$

where $\sigma_{A_{1}^{W}}^{2}$, $\sigma_{A_{1}^{Q}}^{2}$, $\sigma_{A_{1}^{W}A_{1}^{Q}}$ are the variance components of the first, $\sigma_{A_{2}^{W}}^{2}$, $\sigma_{A_{2}^{Q}}^{2}$, $\sigma_{A_{2}^{W}A_{2}^{Q}}$ are the variance components of the second trait. $\sigma_{A_{1}^{W}A_{2}^{Q}}$ and $\sigma_{A_{2}^{W}A_{1}^{Q}}$ are cross-trait covariances.

From the BLUP solution, the sum of the queen effect and the worker effect of the potential offspring queen is used as the combined genetic effect (raw breeding value) of the colony. The unit is the same as that of the original phenotype.

To support a rational selection decision, the raw breeding values are normalized with respect to the breeding population, which is the set of 36,270 tested queens of the last 5 years. The raw breeding values are linearly transformed so that the breeding population forms a distribution with average 100 and standard deviation of 10, to produce the breeding values published in BeeBreed [26].

The breeding values of hygienic behavior and VID are normalized with respect to those 22,210 colonies in the last 5 testing years tested for at least one of the *Varroa* traits. For VID, the normalized breeding values are reversed in that low infestation represents high breeding values.

The breeding values for hygienic behavior and VID are combined with the *Varroa* index at the ratio of 57:43. The share of the VID has been increased from year to year as the *Varroa* measurements were introduced 2006 and coverage and quality of data lagged behind hygienic behavior.

Inbreeding coefficients are calculated with PInCo as diagonal entries of the relationship-inbreeding coefficients [39].

### 2.3. Validation

To assess the quality of the breeding value estimation, prospective breeding values of re-enacted estimations of past years are related to eventual phenotypes. The following algorithm implements this:

1. Iterate reference year *y* from 2014 to 2019.
2. Delete all performance data from test year *y* and later.
3. Calculate normalized breeding values using the full pedigree.
4. Calculate the relative performance by subtracting the apiary effect (from a BLUP calculation with performance data of year *y* included but not later) and add the average apiary effect.
5. Divide the tested colonies into four classes by ranked breeding values.
6. For each of the four classes, calculate the average adjusted performance.
7. Average the result for all years *y*.
8. Calculate the Pearson correlation coefficient of breeding values and adjusted relative performances as an additional parameter.

The significances of the correlations have been computed with the rcorr function of the Hmisc package of the R software environment [52].

## 3. Results

**Figure 1:**
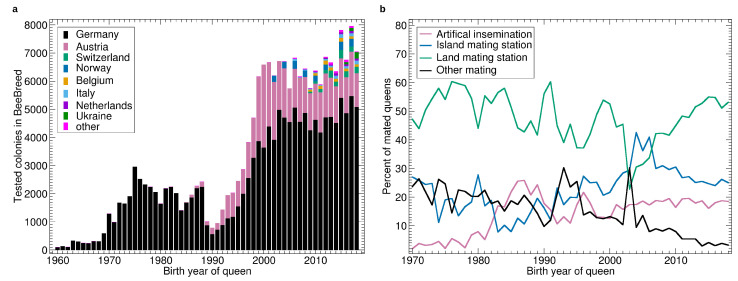
(**a**) Number of tested colonies by year, bars stacked by country. (**b**) Percentage of queens with different types of mating. The category “other mating” refers to race mating stations, apiary mating or unknown paternal ancestry.

The first breeder queen registered in BeeBreed hatched in 1949 but until 1960 only a few queens were recorded. The number gradually increased to about 100 in 1960, and was still low in the 1960s, see Figure 1a. Thereafter, the number increased strongly in the first half of the 1970s to about 2000 queens which remained stable until 1989. In 1990 there was a sharp drop to about 1000 queens followed by a strong increase until 2000, accelerated by the Austrian queens. After 2000, the number of colonies remained stable in Germany and Austria and breeding programs of other countries lead to a slight increase in the total number of queens, resulting in an active breeding population between 7000 and 8000 queens.

See Figure 1b for the ratio of different types of mating. The share of artificial insemination generally increased throughout the study period with some interruptions. In the late 1980s, there was a temporary peak as well as in the late 1990s. Land mating stations were the dominant form of controlled mating with a temporary drop around 2005 when the island mating stations dominated for a limited amount of time. The share of insecure mating generally decreased throughout with some temporary peaks in the mid 1990s and early 2000s.

See Table 5 for the descriptive statistics on the traits. Apiary invariants are the number of measurements and observations which are equal throughout the whole apiary. They are not used in BLUP as they do not contain discriminatory information. The trait VID has a negative mean as it is inverted so that higher values refer to more resistance. Positive values are possible through high mite fall measurements.

See Table 6 for the phenotypical correlations between traits. Not surprisingly, the largest correlation is between gentleness and calmness, albeit lower than the genetic correlation. Similar to the genetic correlations, all correlations are positive. The lowest correlations are for VID to the classical four traits.

### 3.1. Breeding Progress

**Figure 2:**
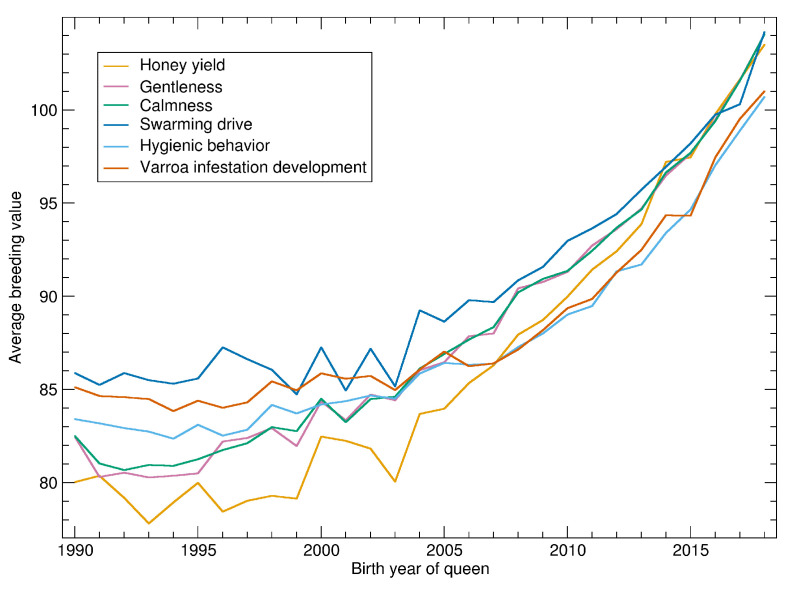
Yearly averages of normalized breeding values for all tested queens.

See Figure 2 for the progression of normalized breeding values calculated on 14 February 2020. There is little progression until about 2000 when the breeding values started to become more relevant in the breeding decisions. After 2005 there is a consistent and accelerating progression in all traits.

As breeding values shown in Figure 2 are normalized with respect to the population, their slope can be compared. For the relative increase, the traits can be ordered as: honey yield, gentleness, calmness, swarming, hygienic behavior and VID.

Breeding values are normalized to the standard deviation of 10. Thus, the time needed to improve the population by one standard deviation can be estimated. For honey yield, the average breeding values of 90 in 2010 was increased to 100 in 2016. Thus, 6 years were needed to increase from 90 to 100. For the other traits, the time was up to 10 years, depending on which years were compared. In the final years, the time seems to be shorter but this has to be considered with caution as the reliability of the recent breeding values is relatively low.

For hygienic behavior and VID the averages are below 100 because the normalization is restricted to colonies where these traits are measured. Thus, the low increase of *Varroa*-related traits is partly explained by the fact that the traits are only measured in about half of the population. In the subpopulation of all assessed colonies, the increase of hygienic behavior is highest (not shown).

### 3.2. Honey Yield

**Figure 3:**
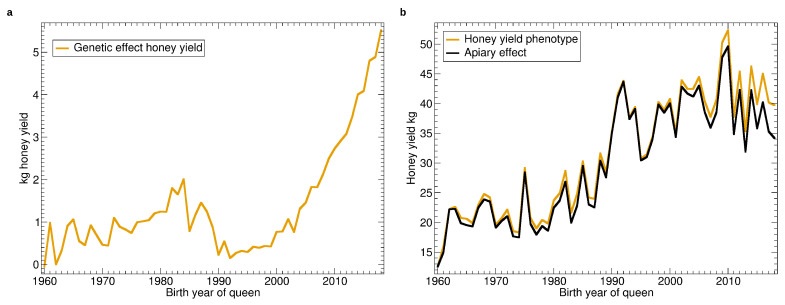
Yearly averages of honey yield: (**a**) Progression of the genetic effect. (**b**) Progression of the average apiary effect and phenotype as the sum of genetic and apiary effect.

See Figure 3 for the yearly averages of genetic and apiary effect as well as its sum—the phenotype. As can be seen from Figure 3b, the apiary effect, respectively the environmental conditions, varied strongly from year to year, which can be attributed to the seasonal weather. From the viewpoint of the beekeeper, when comparing the average honey yield from year to year, it is difficult to observe the genetic effect.

There is a large positive trend of the fixed effect which can be attributed to colony management which is directed at higher per-colony yield. In the last decade this positive trend appears to have turned. The genetic effect slowly increased from 1960 to 1985 (Figure 3b) for a total of about 2 kg improvement. The improvement vanished in the next years, and another increase started in 1992 which intensified in 2004 to reach a total of over 5 kg comparing 1992 with 2018.

**Figure 4:**
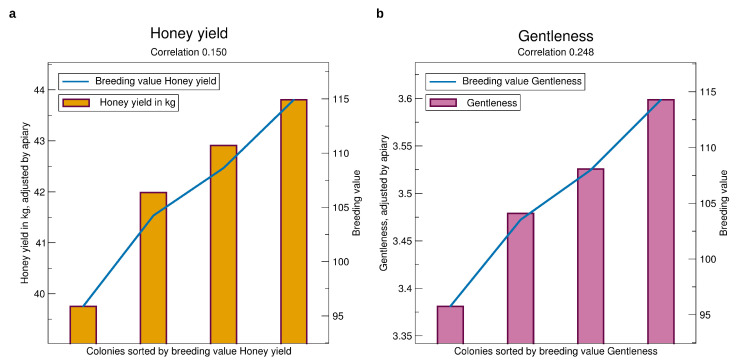
Comparison of prospective breeding values of queens tested in a year, iterated from 2014 to 2019, to the phenotypes standardized to an average apiary, see Section 2.3. The bars represent the adjusted average performance for the colonies, split into four sets by sorted breeding values, quantified by the y-axis to the right. The blue line represents the average breeding values quantified to the y-axis to the left. The x-axis refers to colonies sorted by breeding values. Thus, the breeding values (blue line) are displayed as a line—although for clarity the average for each of the classes is shown. (**a**) Honey yield, (**b**) Gentleness. Significances of all correlations are $p<10^{-16}$. The standard errors are about 10 kg for honey yield and 0.4 for gentleness. They are not displayed to avoid compression of the y-scale.

See Figure 4a for the validation results which can be interpreted as the honey yield one can expect on an average test apiary after selecting from colonies in a certain breeding value range. Selecting a colony of the 25% best breeding values, one can expect a yield of 43.5 kg, while selecting the bottom 25%, the expectation is only 38.5 kg. Comparing the top 25% with the next highest quarter, there is a difference of 0.8 kg, where the average breeding values are 115 and 107, resp. The expected honey yield of the lowest quarter has a large gap of 2 kg to the next highest quarter. Thus, the population has an asymmetric distribution in this respect.

The correlation of the breeding values calculated before knowing the phenotype with the phenotype finally observed is 0.150. Not suprisingly, it is highly significant because it is based on 26,549 colonies. A phenotype cannot be accurately predicted from the genotype, also indicated by the heritability of 0.14, but the reliability of prediction is sufficient for effective selection.

### 3.3. Manageability Traits

**Figure 5:**
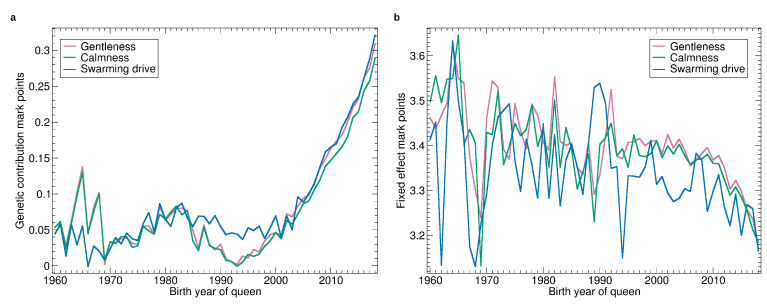
Yearly averages of gentleness, calmness, and swarming drive: (**a**) Progression of the genetic effect. (**b**) Progression of the average apiary effect. The numbers refer to the marks, ranging from 1 (worst) to 4 best. Their differences are called mark points, e.g., the difference between the mark 3.8 and the mark 3.4 are 0.4 mark points.

See Figure 5 for the progression of the manageability traits in absolute terms. The general pattern is similar to that of honey yield, little progress until 1985, then a drop until the early 1990s, and a strong increase afterwards. The drop affected gentleness and calmness much more than swarming drive.

The increase in marks has reached 0.3 when comparing 1995 with 2018 which is remarkable as the mark ranges from 1 to 4 and the colonies considered as well-manageable are usually evaluated with marks from 3 to 4.

The fixed effects varied widely without any trend until 2000 and decreased constantly since. The particular wide deviations of the apiary effect of swarming reflect very different swarming conditions from year to year. The fixed effects dropped by 0.2 when comparing 2008 with 2018 which indicates that the requirements for colonies to get good marks increased. This underlines that the perception of the properties of colonies is currently changing.

**Figure 6:**
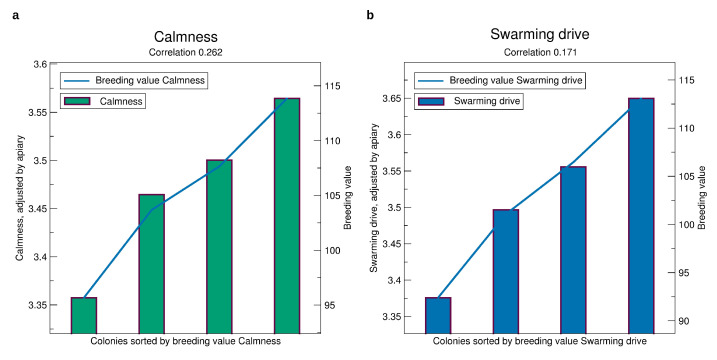
Comparison of prospective breeding values of queens tested in a year, iterated from 2014 to 2019, to the phenotypes standardized to an average apiary, see Section 2.3. (**a**) Calmness. (**b**) Swarming drive. Significances of all correlations are $p<10^{-16}$. The standard errors are about 0.4 for calmness and 0.6 for swarming.

See Figure 4b for the validation of the breeding values of gentleness. Selecting a colony among those with the best 25% breeding values one can expect a mark of nearly 3.6, whereas selecting a colony from the bottom 25% the expectation is only 3.35. For calmness, Figure 6a, the finding is similar. For swarming drive, Figure 6b, the difference is even higher at about 0.3 mark points.

The correlation of predicted phenotype to phenotype for the gentleness and and calmness is about 0.25 which is much larger than for honey yield. For swarming drive, it is 0.17 which is still larger than for honey yield which is remarkable due to the fact that in quite a few apiaries and years colonies can not be distinguished in their swarming behavior because the seasonal weather is not prone to swarming.

### 3.4. *Varroa* Resistance Traits

**Figure 7:**
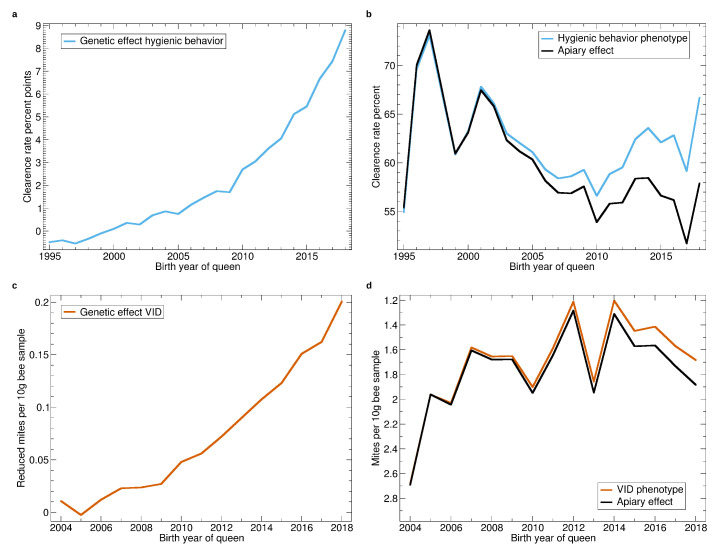
Yearly averages of hygienic behavior and *Varroa* infestation development (VID). (**a**) Progression of the genetic effect for hygienic behavior. (**b**) Progression of the average apiary effect and phenotype for hygienic behavior. (**c**) Progression of the genetic effect for VID. The trait is inverted, thus, higher values represent improved resistance. (**d**) Progression of the average apiary effect and phenotype for VID. The y-axis is reversed because low infestation development values represent improvement.

See Figure 7a,b for genetic and apiary effect of hygienic behavior. Breeding added 9% to the clearance rate. This is a huge effect considering that hygienic behavior is only assessed since 1995, in only a few colonies in the beginning, and still only in about half of the population. The strong breeding progress for hygienic behavior benefits from the high heritability of the trait and the good repeatability of the test [21].

The apiary effect of hygienic behavior (Figure 7b) decreased throughout (except for 1995 where very few colonies were tested) which reflects the fact, that the waiting times for the pintest are now much shorter (down to 3 h) than in 1995, when mostly 24 h have been used.

Breeding for decreased VID (Figure 7c) resulted in a reduction of 0.2 mites with respect to a measurement at the 27th calendar week and 10 g bee sample. This decrease is remarkable as the heritability of the trait is relatively low (see Table 3) and it is only assessed since 2003. However, comparing it to an average phenotype of 2 (Figure 7c) shows that there is still a long way to go for a sufficient *Varroa* resistance.

**Figure 8:**
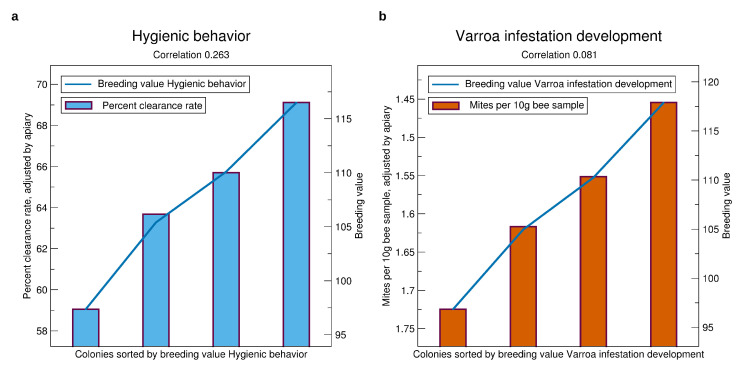
Comparison of prospective breeding values of queens tested in a year, iterated from 2014 to 2019, to the phenotypes standardized to an average apiary, see Section 2.3. (**a**) Hygienic behavior, (**b**) VID. Significances of correlations for hygienic behavior are $p<10^{-16}$, for VID p<0.0037. The standard errors are about 15 percent points in clearance rate and 1.5 for VID.

See Figure 8 for the validation results of the *Varroa*-related traits. Generally, there is a considerable selection advantage of the queens with high breeding values. Comparing the average of the highest quarter with the lowest quarter, the expectation is 10% higher clearance rate in the pintest, and 0.25 less mites per 10 g bee sample.

The correlation of predicted to real phenotypes is much higher for hygienic behavior (highest of all traits) than for VID. This corresponds well with the low heritability of VID.

### 3.5. Inbreeding

**Figure 9:**
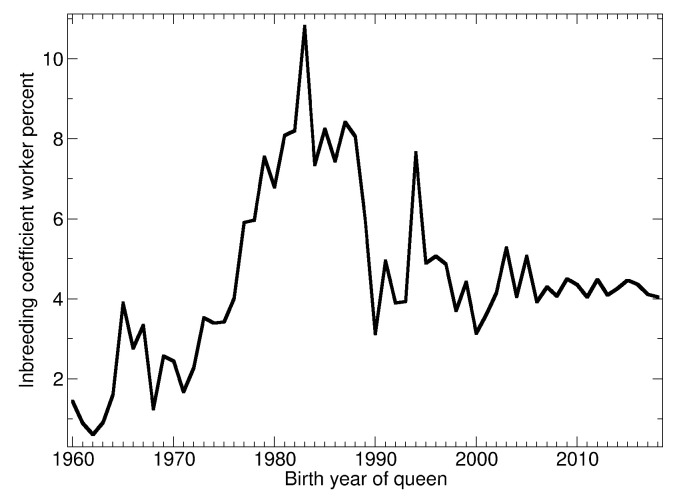
Average inbreeding coefficient of the workers by year.

See Figure 9 for the yearly average of the inbreeding coefficient. Before 1975 the coefficient was low, increased around 1980 to up to 10%, fell until 2000 and remained in a narrow range about 4% since.

## 4. Discussion

### 4.1. Breeding Progress

In a retrospective analysis, the past breeding progress of the registered *A. m. carnica* population, which amounts to roughly 5 kg more yield per colony, about 0.3 mark points of manageability properties, 9% of clearance rate and 0.2 less mites per 10 g bee sample, has been predominantly realized after 2000, when the breeding value estimation took effect.

The yearly genetic improvement in absolute terms (independent from the normalization basis), obtained from a linear regression on queens from 2009–2018, is 336 g for honey yield, 0.017 mark points for gentleness and for calmness, 0.018 mark points for swarming, 0.73 percent points for hygienic behavior and a reduction of 0.018 mites per 10g bee sample for VID.

In a previous publication, the genetic improvement for the *A. m. carnica* population has been estimated at 0.59% per year for honey yield and 0.44% per year for gentleness for the time span 1995–2006 on the normalization basis of queens of 2001–2005, as compared to the time span 1970–1989, where it is 0.11% and 0.01%, respectively [53]. In 2016, the yearly genetic improvement has been reported as 0.61% for honey yield, 0.42% for gentleness, 0.43% for swarming tendency, and 0.60% for the Varroa index on the normalization basis of queens of 2010–2014 [54]. Here, we calculated percentages in the same way for comparison. The yearly genetic improvement with respect to the current breeding population of birth years 2014–2018 (relative improvement as represented by normalized breeding values), averaged over the time span 2009–2018 is 1.67% for honey yield, 1.45% for gentleness, 1.45% for calmness, 1.26% for swarming, and 1.47% for the Varroa index. When compared to the time span 1995–2006, the yearly increase is 0.53% for honey yield and 0.56% for gentleness has roughly tripled. Thus, the previously reported breeding progress acceleration has been surpassed by far in recent years. In this report years, 1990–1994, have not been removed from the analysis unlike previous reports [53,54] because the relative impact on the analysis is much less as the majority of colonies is tested after 2000.

Intriguingly, until 1990 the increase of honey yield is fully covered by the apiary effect Figure 3b while there is nearly no increase in the genetic effect, see Figure 3a. This is explained by the restructuring in the honeybee breeding in the early 1990s years where the introduction of breeding values estimation played a large role. Until 1990, an important breeding goal in Germany and Austria beside the colony’s properties was the maintenance of breeding lines [55], targeting the heterosis effect of the crossing of lines [56]. In the 1990s, there was a paradigm shift where the performance of the breeding colony and the inbreeding came into focus [57].

Before 1990, colonies were mainly selected by the relative performance (compared to the apiary average) which insufficiently expressed genetic value. A large disadvantage of selection by own performance is that the direct effect is overemphasized which is counterproductive when genetic correlation with the maternal effect is highly negative [30]. With the introduction of breeding value estimation, the breeders responsible for a mating station began to equip the mating station with drone-producing colonies descending from a highly valued sire queen instead of taking compromises to ensure the purity of the breeding line which lowered the genetic progress because of the large impact of sires on the population.

Before 1990, there was a large breeding population in the former GDR. After 1990, subsidies for breeding were terminated [58]. Thus, these breeds have largely been discontinued, and the breeding population decreased dramatically, which can also be clearly seen in Figure 1a. The centralized and publicly-visible data management in BeeBreed had the effect that gaps or errors in pedigree recording could be detected and corrected. The more the breeders cooperated with administrators and other breeders and used BeeBreed functions like Breeding planning and the Pedigree Browser, the more records were corrected using the original hand-written records. Thus, the negative effect of colonies with unknown ancestry for the accuracy of breeding values decreased gradually.

The measurement of mite infestation is hampered by some technical factors which include (i) the difficulty to obtain a representative bee sample, (ii) small deviations in humidity strongly affect the efficacy to remove mites applying the powder sugar method, and (iii) a strong effect of the environment on mite population growth and brood development. Thus, the low heritability (Table 3) and the low correlation of breeding values with phenotypes (Figure 8b) is less surprising than the stable upward trend of the VID breeding values. It can be explained by the high genetic correlation with hygienic behavior.

### 4.2. Reasons of Progress

While a positive effect of available breeding values on breeding progress is very likely, it is only one of the factors that have positively affected the breeding progress. The public availability of a central registry of breeders and queens in BeeBreed may have promoted the exchange of breeding material. Furthermore, the public awareness of the relevance of beekeeping has prompted national and international funding of honeybee breeding (e.g., HoneyMoney of the E.U. which is partly used to support breeding) which motivated beekeepers to start breeding leading to the increase of the breeding population. European projects such as SmartBees have contributed to educating beekeepers to become breeders. The efforts of the D.I.B. and ACA to support breeding as a cornerstone in the challenges beekeeping faces have intensified since the 2000s.

Only after 2000, the majority of the *A. m. carnica* breeding population in Germany and Austria is represented in BeeBreed. Thus, the breeding value progression before does not have the same level of certainty. Foreign testing (exchange of queens between breeders) has become more widely implemented since 2000 as a factor for reliability of breeding values. This may have led to a dynamic cycle, where transparency, visibility, selection progress and motivation of the breeders have promoted each other and had an impact towards improved honeybees.

Beekeeping has become more challenging since 1990 due to diseases, environmental change, and agricultural auxiliary substances toxic to bees, not least reflected by colony losses [59]. As can be seen from this report, average honey yield per colony peaked in 2010. This should not be mistaken as a poor transfer of breeding progress into field performance. Improving genetics is not primarily directed to improve the honey bee performance but became a necessity to even keep the status of vitality and colony health which allows a sufficient honey yield.

While the stagnation of honey yield phenotype may need some explanation, for the manageability traits it is straightforward. Slowly changing properties of the bees will lead to a changing evaluation yardstick. The same is true for the pintest, where the waiting times have been successively reduced from 24 h to 3 h, resulting in a changing baseline of evaluation. The baseline for mite infestation development is also constantly changing by beekeeping procedures. While mites become more and more wide-spread, the partial resistance may prompt breeders to reduce the chemical treatments which may lead to higher mite infestations etc. In conclusion, the effect of selection cannot be directly seen on the phenotype. Only the separation of the genetic effect from the apiary effect makes the changes clearly visible.

Both genetic and phenotypical correlations are positive between all traits. The general agreement among most breeders to improve all traits simultaneously avoided breeding stock which developed one trait at the the expense of the other. This is reflected in the concurrent genetic progress of all traits.

*Varroa* resistance is occasionally associated with aggressiveness [60] and breeding for resistance often initially ignores other traits [61]. However, this does not appear to be relevant to the whole breeding population or compensated by the problems low resistant colonies have with the burden of *Varroa* infestation.

### 4.3. Inbreeding

Before 1975 and in the early 1990s the recorded pedigrees were often not complete and contained gaps and errors. Thus, the inbreeding coefficients estimated from the pedigree may be lower than the real inbreeding. In the 1980s and after 1994 the pedigrees are sufficiently complete that the estimates can be considered as reliable.

The high inbreeding coefficients in the 1980s can be seen as the result of the predominant line-breeding in that time, where the population was divided into subpopulations with little exchange [62]. Adverse effects of inbreeding have been observed [63] and could be traced back to the advent of a central register of queens in the 1990s and finally the web service BeeBreed. Thus, low inbreeding became one of the breeding goals, simplified by precalculated inbreeding coefficients of potential offspring available in BeeBreed from 2005. Breeders can now select a mating station or drone producing colonies for artificial insemination not only for the breeding values but also the expected inbreeding. By not mating closely related animals, genetic bottlenecks are reduced and genetic diversity is possible also in dam/sire combinations.

Consistent with simulation studies [64], the *A. m. carnica* population follows the development of a stable breeding progress with a very limited increase in inbreeding.

### 4.4. Dangers of Breeding Progress

For all traits, the results indicate accelerated breeding progress. Is there a danger of genetic depletion? The genetic potential of the *A. m. carnica* population is still largely unknown. In the first half of the 20th century, there was a mixture of different bee subspecies in Germany, many of them hybridized and with problematic properties. In the effort to improve the quality of honeybees since 1950, in Germany and Austria the strategy pure breeding of *A. m. carnica* (and *A. m. mellifera*) has been adopted based on (i) controlled mating at mating stations, (ii) phenotypical analysis to select for race-typical bees, and (iii) importation from the original distribution area. While the former two decrease the genetic diversity, the latter increases it. Since the total *A. m. carnica* population in Europe is large, its diversity can only be assessed with genomic analysis [65]. However, the large breeding progress at no observable increase of inbreeding indicates a sufficient genetic diversity.

The breeding progress in *A. m. carnica* increases the attractiveness for beekeepers and gives rise to the risk of losing genetic diversity [54] and endangering other subspecies and species [66]. However, adaptation to the environment is an equally important prerequisite for successful beekeeping and it has been found that imported breeds lose desirable properties if used in a different environment [67,68]. The discontent with the properties of endemic subspecies enticed local beekeepers to import bees [69], and the potential loss of the local subspecies obstructs the passage to restart with regionally adapted bees when diseases and colony losses among the imported bees would make this necessary. Climate change will eventually force beekeepers to break new ground, and it will be invaluable if breeders have bees at hand which tolerate heat and drought. Thus, improving the regionally adapted honeybees in a similar way as *A. m. carnica* has been recognized as the more sustainable strategy compared to importing highly valued *A. m. carnica* [70].

## Figures and Tables

**Table 1:** Overview over datasets as of 14 February 2020, date of data closure.

Category	Data Sets
Number of queens referenced in database	224,081
Number of queens with explicit information (datasets)	217,860
Number of queens subjected to a performance test [16]	195,458
Number of queens tested for honey yield, gentleness, calmness and swarming	156,117
Number of queens with additional measurement of *Varroa* traits	37,175
Number of queens without ancestors (base population)	8761

**Table 2:** Total number of queens subjected to performance test by country.

Country	Tested Queens
Germany	147,449
Austria	39,910
Norway	1926
Switzerland	1728
Belgium	1044
Italy	917
Netherlands	467
Ukraine	422
Sweden	343
France	188
Luxembourg	60
Croatia	6

**Table 3:** Heritabilities and their standard errors of models used in breeding value estimation.

	Honey Yield	Gentleness	Calmness	Swarming	Hygiene	VID
Queen	0.231 ± 0.031	0.202 ± 0.032	0.171 ± 0.028	0.222 ± 0.036	0.122 ± 0.030	0.064 ± 0.019
Worker	0.423 ± 0.051	0.906 ± 0.072	0.857 ± 0.068	0.444 ± 0.060	0.504 ± 0.069	0.039 ± 0.020
Selection criterion	0.341 ± 0.034	0.683 ± 0.043	0.684 ± 0.041	0.327 ± 0.038	0.517 ± 0.047	0.111 ± 0.023
Accessible	0.140 ± 0.014	0.281 ± 0.018	0.281 ± 0.017	0.135 ± 0.016	0.213 ± 0.019	0.046 ± 0.009

**Table 4:** Genetic correlations and their standard errors.

	Gentleness	Calmness	Swarming	VID
Honey yield	0.319 ± 0.057	0.395 ± 0.052	0.148 ± 0.079	
Gentleness		0.885 ± 0.015	0.309 ± 0.065	
Calmness			0.305 ± 0.062	
Hygiene				0.484 ± 0.097

**Table 5:** Descriptive statistics for honey yield, defensive behavior, swarming behavior and *Varroa* resistance traits.

Trait	N	Min.	Max.	Mean	SD	Apiary Invariants	Outliers Corrected
Honey yield	194,461	0	205.5	37.3	23.1	2960	1774
Gentleness	161,218	1	4	3.53	0.50	20,419	n.a.
Calmness	160,732	1	4	3.50	0.50	21,857	n.a.
Swarming	157,953	1	4	3.58	0.73	43,809	n.a.
Hygiene	57,415	0	100	62.8	23.3	655	n.a.
VID	45,538	−154.4	6.9	−1.65	2.73	323	872

**Table 6:** Pearson correlations with standard errors between phenotypes corrected for apiary/year effects obtained from the breeding value estimation. Correlation is calculated only from pairs of colonies with five measurements per apiary/year.

	Gentleness	Calmness	Swarming	Hygiene	VID
Honey yield	0.236 ± 0.0027	0.25 ± 0.0027	0.204 ± 0.003	0.195 ± 0.0043	0.0774 ± 0.0048
Gentleness		0.632 ± 0.0022	0.197 ± 0.0031	0.154 ± 0.0043	0.0944 ± 0.0049
Calmness			0.202 ± 0.0031	0.154 ± 0.0043	0.0965 ± 0.0049
Swarming				0.124 ± 0.0049	0.0785 ± 0.0055
Hygiene					0.159 ± 0.0052

## Legal notice

The breeding data remain the property of the breeders and are not disclosed. The use of breeding data for this publication is arranged in the license agreements of BeeBreed.

## Abbreviations

The following abbreviations are used in this manuscript:

BLUP	Best Linear Unbiased Prediction
REML	Restricted maximum likelihood
AIREML	Average information restricted maximum likelihood
DPQ	drone producing queen
GDR	German Democratic Republic
VID	*Varroa* infestation development
E.U.	European union
D.I.B.	Deutscher Imkerbund (German Beekeeper’s association)
ACA	Austrian Carnica Association
